# Near-Complete Genome Sequences of *Rice Yellow Mottle Virus* Isolates from Senegal

**DOI:** 10.1128/MRA.00937-19

**Published:** 2020-01-09

**Authors:** H. Tall, J. Aribi, S. Camara, A. Pinel-Galzi, N. Poulicard, D. Fargette, E. Hébrard

**Affiliations:** a ISRA, Dakar, Sénégal; b IRD, Cirad, UM, IPME, Montpellier, France; KU Leuven

## Abstract

*Rice yellow mottle virus* in Senegal is reported here for the first time. The near-complete genomic sequences of two isolates (Se1 and Se5) were obtained. A comparison with 18 sequences from West Africa revealed a new cluster with an isolate from Gambia, located at a basal position in the phylogenetic tree.

## ANNOUNCEMENT

*Rice yellow mottle virus* (RYMV) is a single-stranded positive-sense RNA virus species of the *Sobemovirus* genus in the *Solemoviridae* family (1). RYMV is the major biotic constraint to rice cultivation in Africa (2). The virus has been reported in most West African countries (3). However, RYMV had never been found in Senegal, an important rice-producing country. In 2018, a survey was conducted in the Anambe Basin, an irrigated area of 5,000 ha in the south of Senegal. Leaf yellowing and mottling symptoms were observed on rice plants. The presence of the virus was confirmed using double-antibody sandwich enzyme-linked immunosorbent assays (DAS-ELISAs) with polyclonal antisera performed as described in reference 4. Mechanical inoculation with leaf extracts from field samples caused typical yellow mottle symptoms on the susceptible rice variety IR64 grown in controlled conditions. Inoculated plants gave positive results in DAS-ELISAs. This is the first report of RYMV in Senegal.

Total RNA collected from three field samples (Se1, Se5, and Se8) was extracted with the GeneJET plant RNA purification kit (Thermo Fisher). The 720-nucleotide (nt)-long coat protein (CP) gene was amplified using reverse transcription-PCR (RT-PCR) with primers 5′-CGCTCAACATCCTTTTCAGGGTAG-3′ and 5′-CAAAGATGGCCAGGAA-3′ (5). Purified PCR products were directly sequenced with internal specific primers using an ABI3730xls platform. Two readings per base (in the 3′-to-5′ and 5′-to-3′ directions) led to sequence accuracy of over 99.9%. The sequences were compared to a set of CP sequences of 45 isolates representative of the RYMV diversity in West Africa using Molecular Evolutionary Genetics Analysis (MEGA) version 6.06 (6). Isolates from Senegal are closely related (nucleotide identity, >99%). These isolates clustered within a monophyletic group with the isolates from Gambia collected ca. 100 km to the west (GenBank accession no. AM765810, AM765811, AM765812, and AM765813) (7). Interestingly, with a nucleotide divergence of over 4% between isolates from Gambia and from the south of Senegal, this group displayed an unexpected high level of genetic diversity.

**FIG 1:**
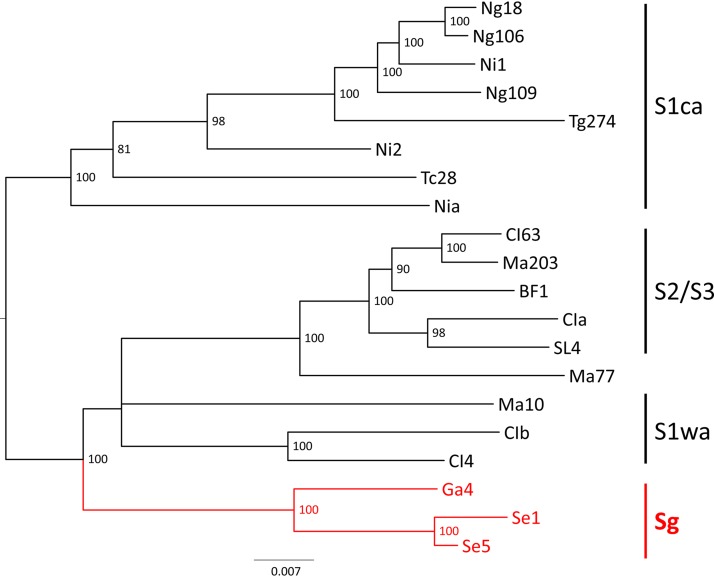
Phylogenetic tree reconstructed with the maximum likelihood method using the best model (GTR+G+I) from the genomic sequences of 20 RYMV isolates from West Africa. The names of the countries are abbreviated as follows: Burkina Faso, BF; Chad, Tc; Côte d’Ivoire, CI; Gambia, Ga; Mali, Ma; Niger, Ng; Nigeria, Ni; Senegal, Se; Sierra Leone, SL; and Togo, Tg. The accession numbers are AM883059 (BF1), AJ608219 (CIa), L20893 (CIb), AJ608206 (CI4), AJ608207 (CI63), FN432838 (Ga4), AJ608208 (Ma10), AJ608209 (Ma77), FN432840 (Ma203), FN432841 (Ng18), MF784437 (Ng106), MF784438 (Ng109), U23142 (Nia), AJ608212 (Ni1), AJ608213 (Ni2), MN233654 (Se1), MN233655 (Se5), AJ608214 (SL4), FN432837 (Ch28), and MF784441 (Tg274). The names of the strains (S1ca, S2/S3, S1wa, and Sg) are given on the right side of the figure. The tips and the branches of the strain Senegal-Gambian (Sg) are colored red. The bootstrap support of the branches (when >70%) is indicated.

Near-complete genomes of two isolates from Senegal (Se1 and Se5) were amplified using RT-PCR with two pairs of overlapping primers (A_S_ and B_AS_ and C_S_ and D_AS_, respectively) using total RNA as a template (Table 1) (8). Primers A_S_ and D_AS_, with 21- and 23-nt lengths, respectively, are located at the 5′ and 3′ extremities of the genome, respectively, meaning that in the genomic sequences, only the nucleotides corresponding to these primers are missing. Other sequencing primers were used to complete the RYMV genomes (Table 1). The sequences were compared to the 18 published full-length sequences from West Africa, including one sequence from Gambia, using MEGA (6) and Recombination Detection Program (RDP4) version 4.94 (9). The two near-complete genome sequences of RYMV isolated from Senegal were 4,450 nucleotides long with a mean G+C content of 54.6%. The RYMV genome is organized into five open reading frames (ORFs) as previously described (1). No recombination events were detected. The two isolates from Senegal are genetically close together (98.9% nucleotide identity) and related to the isolate from Gambia (ca. 96.6% nucleotide identity) (7). The phylogenetic reconstruction showed that the isolates from the south of Senegal and Gambia formed a monophyletic group, named strain Sg, located at a basal position in the phylogenetic tree (Fig. 1).

**TABLE 1:** Primers used in RT-PCR to amplify and sequence the full genome of *Rice yellow mottle virus*

Primer name^*a*^	Positions	Primer sequence	Product size (nt)
A_S_	2–21	5′-CAATTGAAGCTAGGAAAGGAG-3′	2,422
B_AS_	2401–2424	5′-ACTTCGCCGGTTTCGCAGAGGATT-3′	
C_S_	2138–2157	5′-CATGCTGGGAAAAGTGTCTG-3′	2,314
D_AS_	4430–4452	5′-CTCCCCCACCCATCCCGAGAATT-3′	
R5_S_	627–648	5′-GGTCGCTTTCTCACTCGCACC-3′	
R9_S_	1506–1525	5′-ATAGGTGCTGCGGATGGTTA-3′	
R10_AS_	1840–1821	5′-GCTACGGGATGCGATGTCTC-3′	
R11_S_	1875–1895	5′-AAGCGCGTTGAGCAGTTCGT-3′	
R15_S_	2579–2599	5′-AGGGAGCTGGTAGAGAAAGG-3′	
3577bis_AS_	3557–3577	5′-GGCCAGGTGTTAGAAGATAG-3′	
recle_S_	3766–3786	5′-TTACCTCCCTGAGGTGAGCG-3′	
R19_S_	3859–3879	5′-AAGATGAGCAGGACGGCGGG-3′	
RYMVIII_S_	3442–3457	5′-CAAAGATGGCCAGGAA-3′	
RYMVM_AS_	4207–4231	5′-CGCTCAACATCCTTTTCAGGGTAG-3′	

a Sense and antisense primers are indicated by subscript S and AS, respectively.

### Data availability.

The complete sequences of isolates Se1 and Se5 have been deposited in GenBank under the accession no. MN233654 and MN233655, respectively. The ORF3 sequences of isolates Se1, Se5, and Se8 have been deposited in GenBank under the accession no. MH699981, MH699982, and MH699983, respectively.
